# Impact of bio-organic amendment supplemented with phosphate-solubilizing bacteria and arbuscular mycorrhizal fungi on sugarcane cultivation

**DOI:** 10.1038/s41598-025-24805-y

**Published:** 2025-11-20

**Authors:** Suchat Juntahum, Thomas W. Kuyper, Jindarat Ekprasert, Sophon Boonlue

**Affiliations:** 1 Department of Microbiology, Faculty of Science, Khon Kaen University, Khon Kaen, 40002 Thailand; 2 GMS Food, Energy, Water Security Research Institute, Khon Kaen University, Khon Kaen, 40002 Thailand; 3 Postharvest Technology Innovation Center, Science, Research and Innovation Promotion and Utilization Division, Office of the Ministry of Higher Education, Bangkok, 10400 Thailand; 4 Soil Biology Group, Wageningen University & Research, P.O. Box 47, 6700 AA Wageningen, The Netherlands

**Keywords:** Arbuscular mycorrhizal fungi, Phosphate-solubilizing bacteria, Sugarcane, Productivity, Organic farming, Microbiology, Plant sciences, Environmental sciences

## Abstract

The enhancement of soil fertility and promotion of microbial activity are critical for improving sugarcane production. Bio-organic amendments supplemented with arbuscular mycorrhizal fungi (AMF) and phosphate-solubilizing bacteria (PSB) have the potential to sustainably increase cane yield, but limited information exists on their combined action. This study aimed to evaluate their effects on sugarcane cultivation under field conditions. The experiment was carried out in a randomized complete block design with six treatments: T1 (control), T2 (PSB inoculation), T3 (AMF inoculation), T4 (filter cake compost), T5 (mineral fertilizer), and T6 (filter cake compost supplemented with AMF and PSB). Across the sampling periods, mycorrhizal colonization (T3) and microbial activity (T2) averaged 2.1 − and 1.4 − fold above the control, respectively, while the organic amendment (T4) increased cane yield to 36.3 t ha^−1^ with improved soil–microbial–agronomic attributes. However, the effects of single inoculations or compost remained lower than those of mineral fertilization. T6 achieved the highest yield (48.8 t ha^−1^), 2.5 − fold above the control and comparable to mineral fertilization (48.3 t ha^−1^), while also enhancing AMF colonization, FDA hydrolysis, and rhizosphere soluble P. This approach provides sugarcane field-based evidence supporting bio-organic amendments as a sustainable alternative that enhances soil fertility, microbial activity, and productivity.

## Introduction

Sugarcane (*Saccharum* spp.) is a (sub-)tropical crop that requires substantial inputs of both water and nutrients to produce maximum yields. Sugarcane is one of the most important commercial crops in Thailand (the world’s second-largest sugar exporter), especially in Northeast Thailand. Sugar production in Thailand is expected to decline to 13.9 million metric tons annually, marking a second consecutive year of decrease, primarily attributed to drought conditions during the critical vegetative and stalk elongation growth stages^1^. Water shortage leads to a decrease in leaf water potential and stomatal opening, which in turn downregulates genes related to photosynthesis and reduces the availability of plant-CO_2_^2^. Water shortages have a notable impact on cane yield^3^. Insufficient nutrients can also have adverse effects on both plant productivity and cane juice quality^4^. Hence, two major obstacles for sugarcane growers in Northeast Thailand are inadequate water supply and low soil fertility, resulting in an undesirably low sugarcane yield. Therefore, it is important to investigate ways to increase sugarcane production under unfavorable conditions.

Phosphorus (P) plays an important role in enhancing cane yield and juice quality^5^. However, the world might be running short of phosphate ore for mineral-fertilizer production due to the rapid use of non-renewable P resources. There is no atmospheric source that can make P biologically available. Moreover, the orthophosphate anion, the only form of P that plants can take up, is subject to strong sorption reactions with the mineral-soil matrix, thereby making most of the soil P unavailable for plant growth. Consequently, plants can access 3–18% of adsorbed P, with availability depending on the type of soil mineral and P form^6^. To increase P cycling in soil systems, microorganisms capable of P utilization have been used to promote plant growth under environmentally friendly cultivation.

Arbuscular mycorrhizal fungi (AMF) and phosphate-solubilizing bacteria (PSB) are used for their ability to enhance P acquisition through scavenging and/or mining for the cultivation of many crops, including sugarcane. AMF play an important role in enhancing the uptake of nutrients, particularly P, through their extensive extraradical hyphal network^7–9^ and in improving the tolerance of plants to heavy metals and drought^10,11^. The application of AMF and rock phosphate increased cane yield by 51% compared with the control^12^. Several species of AMF are symbiotically associated with sugarcane^13–15^.

PSB form a specific group of plant growth-promoting bacteria (PGPB) that can increase the availability of and subsequently provide P to plants. These bacteria excrete organic acids, phosphatase enzymes, or polymeric substances that dissolve insoluble P into soluble, plant-available orthophosphate, which can be taken up by AMF and plants^16^. The PSB, *Enterobacter cloacae*, improves P uptake efficiency by altering root morphology and enhancing P solubility^17^. Aye et al.^18^ reported that applying mineral fertilizer along with PSB *Bacillus subtilis* resulted in higher available P levels and enhanced sugarcane yield compared with mineral fertilizer only. These findings strongly suggest that PSB is beneficial for the growth and yield of sugarcane. However, mixed results have been reported. PSB and nitrogen-fixing bacteria, including *Pantoea dispersa*, have been reported to be opportunistic pathogens in certain plant species. However, accumulating evidence indicates that *P. dispersa* is not typically pathogenic to sugarcane plants. Instead, it has been shown to promote sugarcane growth by enhancing nitrogen (N) uptake, inducing defense-related gene expression, and detoxifying toxins produced by *Xanthomonas albilineans*, the causal agent of leaf scald disease^19,20^.

To provide more P for plant growth, the application of AMF, which increases the acquisition of available P, combined with PSB, which increases P availability through the solubilization of mineral P, could be considered. Several studies on the co-inoculation of AMF and PSB have shown a larger effect than a single inoculation. For example, co-inoculation of the PSB *Klebsiella variicola* and AMF *Claroideoglomus claroideum* or *Rhizophagus intraradices* significantly increased plant growth and tuber inulin content in sunchoke (*Helianthus tuberosus*) compared with unfertilized and fertilized controls^21^. The biomass of tomato (*Solanum lycopersicum*) was significantly higher in treatments with a combination of AMF and PSB than in single inoculations^22^. Hence, understanding the impact of co-inoculation with both AMF and PSB can aid in enhancing the use of a large pool of soil P.

Compost is nutrient-rich, and the application of compost can increase crop yields and improve biological, chemical, and physical soil properties^23^. Filter cake is a sugar industry waste that contains high amounts of P and N^24^. Approximately 40 kg of filter cake is generated per 1000 kg of ground sugarcane^25^. Filter cake has been used as a fertilizer for compost production in sugarcane cultivation^26^. The application of compost resulted in a better performance when inoculation with either PSB or AMF was applied simultaneously. The application of compost and inoculation with PSB *Bacillus* sp. increased the P concentration in the cane shoots compared with non-inoculated treatments^27^. Similarly, the application of compost with AMF benefitted growth, biomass accumulation, and nutrient uptake^28^. However, the effects of compost application and co-inoculation with both AMF and PSB have not yet been reported in sugarcane. In addition, most experiments have been conducted in controlled greenhouse environments, making it important to evaluate these effects under field conditions. The application of compost supplemented with AMF and PSB may be a sustainable strategy to improve soil fertility and increase the efficiency with which P is acquired and used by plants. Therefore, this study aimed to investigate the efficiency of compost supplemented with AMF and PSB in promoting the growth and productivity of sugarcane in a field trial. The effects of these components on the chemical and biological properties of soil were also investigated.

## Results

### Microbial responses to bio-organic amendments

**Fig. 1:**
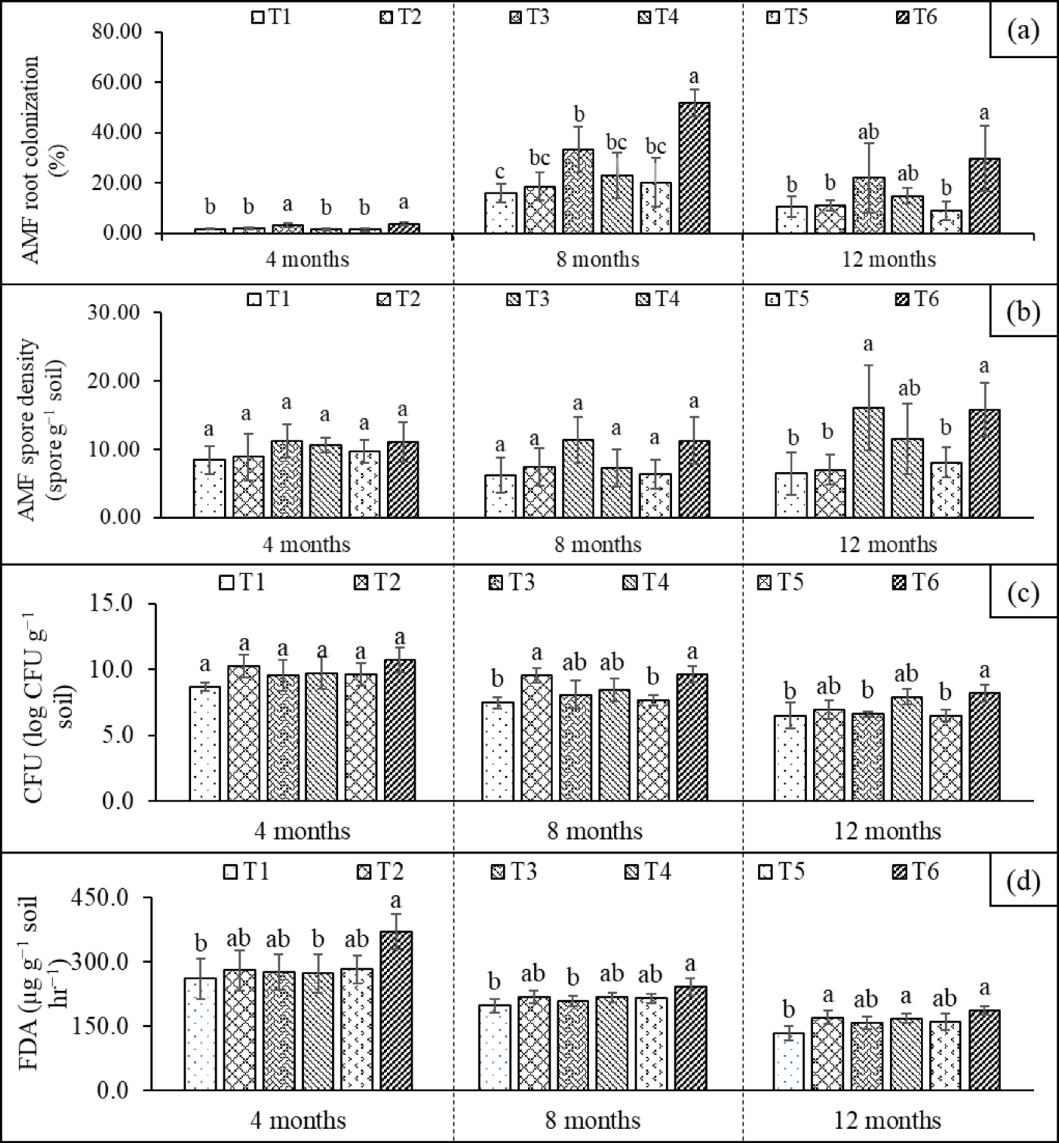
Soil microbial dynamics: AMF root colonization (a), AMF spore abundance (b), bacterial numbers (c), and microbial activity (d) measured at 4, 8, and 12 months under different treatments (T1, uninoculated plants; T2, plants inoculated with PSB (*P. dispersa*; T3, plants inoculated with AMF (*R. clarus*); T4, plants with the addition of an organic amendment (filter cake compost); T5, plants with the addition of mineral fertilizer; T6, plants with the addition of filter cake compost mixed with AMF and PSB). Different letters indicate significant differences (*P* ≤ 0.05) among treatments across each sampling time. Error bars indicate standard deviation.

AMF colonization, which was characterized by the presence of hyphae, arbuscules, and vesicles, was found in all treatments because the field soils were not sterilized. AMF colonization and spore abundance were directly affected by AMF application (T3 and T6) (Fig. 1a–b**)**. The mycorrhizal species found in all treatments included *Glomus* spp., *Acaulospora* sp., and *Gigaspora* sp. No *R. clarus* KKU-F3 spores were observed in the non–inoculated treatments. Very low AMF colonization was observed at 4 months (2–4%), while spore abundance ranged from 8 to 11 spores g^−1^ soil, with the highest values in AMF-applied treatments (T3 and T6). At 8 months, AMF colonization peaked (16–51%), with T6 showing significantly higher values than the other treatments, followed by T3, likely because of the combined effect of compost. However, at 12 months, colonization levels declined markedly (2–13%), although T6 still maintained higher colonization than the other treatments. Notably, the positive effect of compost was not evident at this stage as no significant differences were observed between T3 and T6, suggesting that the interaction between compost and AMF may have been overestimated. Furthermore, the presence of compost and PSB appeared to have no effect on AMF colonization. Spore numbers increased slightly from 8 months (6–12 spores g^−1^ soil) to 12 months (6–16 spores g^−1^ soil), particularly in T6 and T3. These values were significantly higher than those observed in the control (T1) and mineral fertilizer treatments (T5), indicating that AMF application enhanced sporulation.

Both inoculation with PSB and the application of compost resulted in higher bacterial populations in T2, T4, and T6 than in the other treatments (Fig. 1c). At 4 months, bacterial populations in the inoculated treatments (T2 and T6) were already higher than those in the uninoculated controls, indicating successful early establishment in the rhizosphere. By 8 months, bacterial populations in the inoculated plots remained stable and significantly exceeded those in the controls, whereas bacterial populations in all treatments showed a slight decline. At 12 months, bacterial populations continuously declined, yet the highest population was still observed in T6 (Fig. 1c). Although PSB were more abundant in inoculated treatments, they did not differ between treatments, and their numbers remained low (< 10^2^ CFU g^−1^) at 12 months (data not shown). Overall, bacterial populations slowly declined over time, with T6 consistently maintaining the highest levels across all growth stages, suggesting that the integrated microbial and organic amendments provided a more favorable environment for bacterial persistence.

Soil microbial activity measured by FDA hydrolysis was highest at 4 months (261–370 µg g^−1^ soil h^−1^), with T6 showing the greatest activity, reflecting enhanced microbial metabolism during the early growth to tillering phases. By 8 months, FDA hydrolysis declined (198–241 µg g^−1^ soil h^−1^) but remained higher in T6, likely supported by compost or bacterial addition. At 12 months, FDA hydrolysis remained low (134–187 µg g^−1^ soil h^−1^), although T6, T4, and T2 maintained slightly higher values than the other treatments (Fig. 1d). Overall, microbial activity was initially high and decreased over time, with certain treatments (particularly T6) sustaining higher enzymatic activities throughout all stages.

### Effect of bio-organic amendments on plant performance

**Fig. 2:**
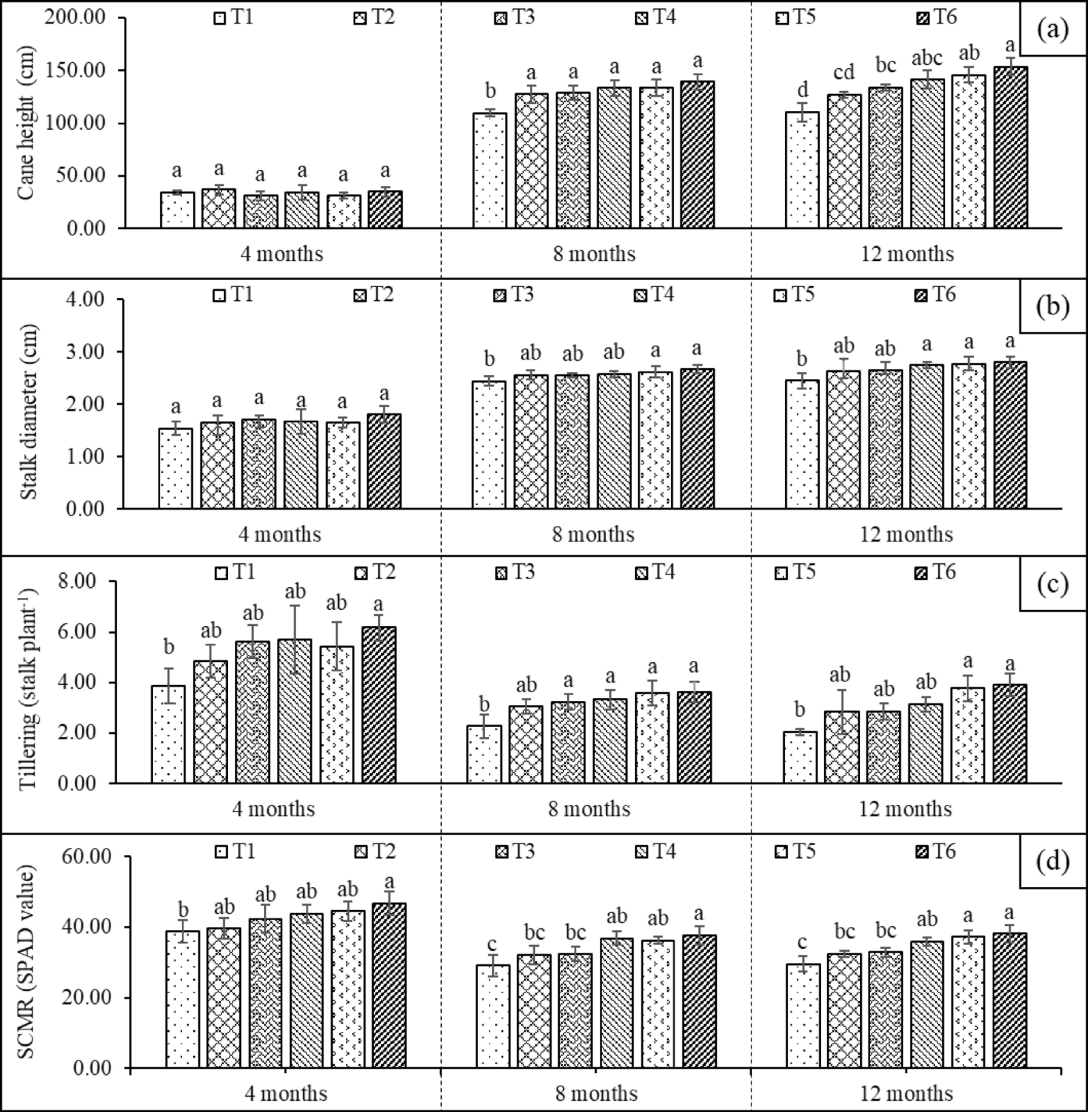
Plant growth performance: cane height (a), cane diameter (b), tillering (c), and SPAD chlorophyll meter reading (SCMR) (d) measured at 4, 8, and 12 months under different treatments (T1, uninoculated plants; T2, plants inoculated with PSB (*Pantoea dispersa*; T3, plants inoculated with AMF (*Rhizophagus clarus*); T4, plants with the addition of an organic amendment (filter cake compost); T5, plants with the addition of mineral fertilizer; T6, plants with the addition of filter cake compost mixed with AMF and PSB). Different letters indicate significant differences (*P* ≤ 0.05) among treatments across each sampling time. Error bars indicate standard deviation.

The physiological traits and growth of sugarcane were enhanced by the application of all treatments compared with the control (Fig. 2). At 4 months (early growth to tillering phase), cane height and diameter ranged from 31 to 37 cm and 1.5–1.8 cm, respectively, with no significant differences among treatments (Fig. 2a–b). Nevertheless, tillering in the control (T1) was significantly lower than T6 (Fig. 2c). Similarly, leaf chlorophyll content (SPAD values) ranged from 39 to 47, with significantly higher values in T6 than in the control (Fig. 2d). At 8 months (grand growth phase), fertilizer application (T4, T5, and T6) strongly influenced growth performance. Although SPAD values declined slightly (29–38), they persisted at significantly higher levels in the nutrient-supplied treatments than in the control (T1). Cane height and diameter increased markedly, ranging from 109 to 139 cm and 2.4–2.6 cm, respectively. Meanwhile, the number of tillers decreased from 4 to 6 at 4 months to 2–4 at 8 months owing to drought-induced mortality. By 12 months (ripening phase), SPAD values remained relatively stable (29–38), yet plants in T5 and T6 maintained higher chlorophyll contents than non-nutrient treatments. Cane height and diameter slightly increased (110–153 cm and 2.4–2.8 cm, respectively), while tillering remained at 2–4 stalks. Stalk mortality was evident in T2 and T3, reinforcing the notion that microbial inoculation alone may be insufficient for sustaining plant performance under drought stress. The highest growth was observed in T6, which did not differ significantly from that of the mineral fertilizer treatment (T5).

### Soil nutrient enhancement by bio-organic amendments

The contents of organic matter, total N, total P, total potassium (K), available P, and exchangeable K in the rhizosphere at harvest are shown in Table 1. The addition of compost significantly increased organic matter content in T4 and T6. Soil with organic amendments or mineral fertilizer applications (T4, T5, and T6) had significantly higher total N and P than the non-fertilized treatments (T2 and T3) and control (T1). Available P significantly increased in T6 and T4 treatments compared with the control (T1). This indicated that P addition through amendments was more effective than inoculation with either PSB or AMF. The highest available P was observed in the T6 treatment. The differences between the control and treatment with PSB or AMF were not significant (Table 1). K exchange significantly increased with the application of compost and mineral fertilizers.

**Table 1:** Nutrient contents (± standard deviation) in the rhizosphere soils of sugarcane plants after 12 months. The treatments: T1, uninoculated plants; T2, plants inoculated with PSB (*Pantoea dispersa*); T3, plants inoculated with AMF (*Rhizophagus clarus*); T4, plants with only the addition of filter cake compost; T5, plants with the addition of mineral fertilizer; T6, plants with the addition of filter cake compost mixed with AMF and PSB. Different letters indicate significant differences (*P* ≤ 0.05) among the data in the same column.

Treatments	Organic matter (g kg^−1^)	Total N (g kg^−1^)	Total P (mg kg^−1^)	Total K (g kg^−1^)	Available P (mg kg^−1^)	Exchange able K (mg kg^−1^)
T1	7.8 ± 1.9 b	0.37 ± 0.10 b	78 ± 21 b	0.89 ± 0.14 a	18 ± 5 c	65 ± 18 b
T2	7.7 ± 1.0 b	0.37 ± 0.11 b	82 ± 16 b	0.96 ± 0.30 a	31 ± 9 c	97 ± 28 ab
T3	7.9 ± 0.8 b	0.37 ± 0.09 b	88 ± 23 b	0.90 ± 0.21 a	31 ± 8 c	94 ± 20 ab
T4	10.6 ± 1.5 a	0.50 ± 0.07 a	198 ± 49 a	1.10 ± 0.18 a	68 ± 17 ab	109 ± 15 a
T5	7.4 ± 1.4 b	0.51 ± 0.07 a	154 ± 43 a	1.09 ± 0.15 a	47 ± 16 bc	115 ± 36 a
T6	12.3 ± 1.1 a	0.52 ± 0.09 a	192 ± 47 a	1.08 ± 0.20 a	85 ± 19 a	112 ± 35 a

### Productivity improvement in sugarcane

Table 2 shows that different treatments significantly influenced the yield components of sugarcane. The uninoculated plants (T1) had the lowest °Brix of 21.5, commercial cane sugar (CCS) (14.1%), and cane yield (19.8 t ha⁻¹). Inoculation with PSB (T2) and AMF (T3) slightly improved these parameters, with the AMF-treated plants showing higher CCS (14.8%) and cane yield (30.3 t ha⁻¹). The application of an organic amendment (T4) further enhanced sugarcane performance, while mineral fertilizer (T5) significantly increased all yield components, particularly cane yield (48.3 t ha⁻¹). The highest values were recorded in the filter cake compost mixed with AMF and PSB treatment (T6), with a °Brix of 23.5, CCS of 15.4%, and a cane yield of 48.8 t ha⁻¹. These values were 2.46 times higher than those of the control (T1), highlighting the combined beneficial effect of these treatments on sugarcane productivity.

**Table 2:** Yield components (± standard deviation) of sugarcane grown after 12 months. Treatments were as follows: T1, uninoculated plants; T2, plants inoculated with PSB (*Pantoea dispersa* KKU-TP3); T3, plants inoculated with AMF (*Rhizophagus Clarus* KKU-F3); T4, plants applied with an organic amendment (filter cake compost); T5, plants treated with mineral fertilizer; T6, plants applied with the filter cake compost mixed with AMF and PSB. Different letters indicate significant differences (*P* ≤ 0.05) among the data in the same column.

Treatments	Sugarcane productivity		
	°Brix	CCS	Cane yield (t ha^−1^)
T1	21.5 ± 0.5 c	14.1 ± 1.0 b	19.8 ± 2.6 e
T2	22.2 ± 0.6 bc	14.5 ± 0.3 ab	28.7 ± 4.1 de
T3	22.3 ± 0.2 bc	14.8 ± 0.5 ab	30.3 ± 1.4 cd
T4	22.8 ± 0.5 ab	15.2 ± 0.4 ab	36.3 ± 2.7 bc
T5	23.4 ± 0.9 a	15.3 ± 0.2 a	48.3 ± 2.7 a
T6	23.5 ± 0.3 a	15.4 ± 0.3 a	48.8 ± 4.5 a

**Fig. 3:**
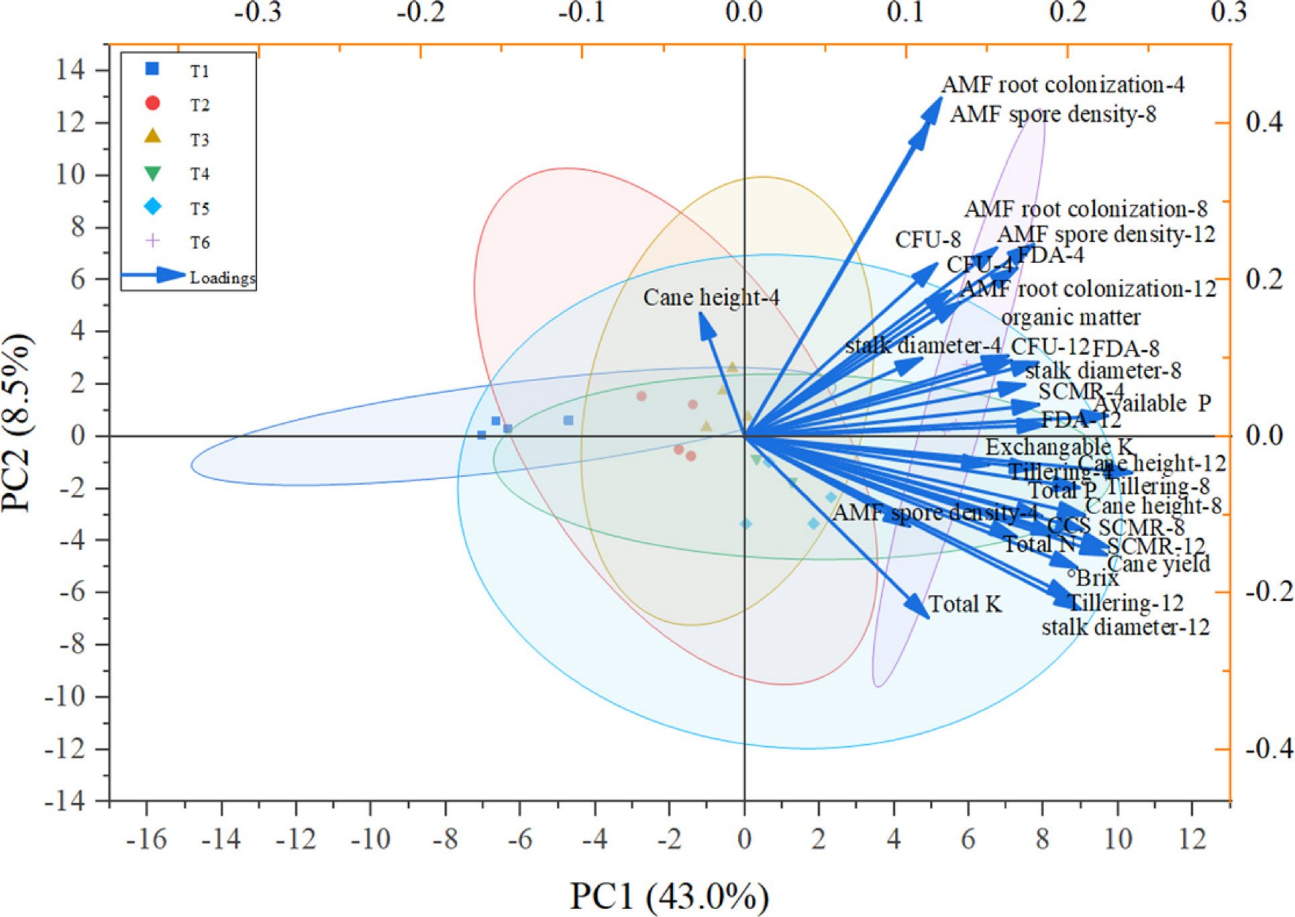
Principal component (PC) analysis biplot integrating soil microbial, plant growth, soil chemical, and productivity variables under different treatment applications.

Principal component analysis (PCA) integrating chemical, biological, and plant biometric variables was performed to obtain an overview of treatment effects (Fig. 3). All variables met the assumptions required for PCA, confirming its suitability for multivariate evaluation. The first two components explained 43.0% (PC1) and 8.5% (PC2) of the total variance. All parameters, except cane height at 4 months, loaded positively on PC1 and clustered together. Treatments receiving fertilizer (T4 and T6), particularly the inoculant combination (T6), scored strongly positive on PC1, indicating concurrent improvements in microbial activity, soil fertility, and plant performance. In contrast, control plots (T1) were mainly located on the negative side of PC1, reflecting lower microbial and plant performance, whereas single-inoculation treatments (T2 and T3) were partially separated from T1, with some T3 replicates positioned on the positive side of PC1, indicating observable improvements compared with the control. The variables contributing most strongly to PC1 were available P, FDA hydrolysis, AMF colonization, and cane yield, indicating that improvements in soil fertility, microbial activity, and plant performance were the main drivers of treatment separation. PC2, which accounted for a smaller proportion of the variance, was mainly influenced by tillering, cane height, and organic matter. Overall, the PCA confirmed that the combined bio-organic amendment (T6) produced the most integrated positive response across soil chemical, microbial, and agronomic variables.

## Discussion

The microbial dynamics were shaped by AMF, PSB inoculation, and compost application (Fig. 1). The integrated use of compost and microbial inocula (T6) enhanced soil microbial function and persistence, thereby supporting improved sugarcane growth. Bacterial populations and microbial activity (FDA hydrolysis) peaked during the early growth stages and gradually declined over time, whereas AMF colonization closely reflected the developmental stages of sugarcane. Notably, T6 showed higher enzymatic activity and microbial persistence than the other treatments.

The positive effect of compost on AMF colonization was not evident, and the PSB population remained low (< 10² CFU g⁻¹) despite inoculation (Fig. 1). In our study, the conversion of paddy fields to sugarcane plantations showed inoculum limitation for AMF, as their abundance in T1 was lower than that in T3 (Fig. 1a-b). An increase in mycorrhizal colonization in sugarcane roots due to mycorrhizal inoculation agreed with previous studies^29,30^. During 4–8 months, the application of AMF combined with compost appeared to stimulate fungal colonization. However, by 12 months, the organic amendment did not further increase the populations of PSB and AMF, as their abundances in T6 were not significantly different from those in T2 or T3. It has frequently been reported that organic amendments favorably affect AMF symbiosis and their activity, leading to increased root colonization, hyphal density, AMF sporulation^31^, and thereby boosting AMF biomass by 78.75%^32^. The lack of an additional effect of compost with AMF inoculation in our study was consistent with the observations of^33^, who reported that the colonization intensity and taxonomic diversity on conventional farms were higher than those on organic farms. The combination of AMF and *Trichoderma* sp., along with the application of organic fish waste to soil, was proposed as a successful strategy for enhancing plant performance, including plant height, root length, number of leaves, leaf area, plant biomass, soil aggregate stability, and soil chitinase activity^34^. The addition of compost significantly increased AMF colonization, AMF growth, and sporulation compared with the non-compost treatment^35^, which is different from the results of our study. One possible explanation is that compost addition improved the physical conditions for AMF, but the P-rich compost negatively affected AMF. In our study, compost application did not enhance AMF colonization, as expected. The strong increases in total and extractable P (Table 1) would have caused a decline in AMF abundance. This could serve as a warning that compost quality, especially the P content of compost, is a critical factor when managing soil biota, such as AMF and PSB in the field. Colonization, which was more effectively induced in restricted and nutrient-poor soil conditions, was inhibited by P fertilization. Downregulation of the mycorrhizal uptake pathway is a plant-mediated response that depends strongly on soil P availability. High P availability can suppress AMF development because plants downregulate the fungal uptake pathway when the P concentration at the root surface exceeds the threshold required for uptake^36^. Mechanistically, when AMF hyphae deplete P near the root, plants suppress their own direct P transporters and shift their reliance to the fungal pathway^9,37^. Conversely, in soils or with organic amendments rich in P, plants reduce the need for AMF, and the mycorrhizal pathway becomes less beneficial or even costly^36,38^. This explains why the co-application of compost and AMF, although often reported to promote AMF colonization, may show no additive effect in some cases. Soussani et al.^39^ reported that the decline in root colonization resulting from the combined application of AMF and compost could be attributed to the high levels of organic matter and nutrients in the soil, particularly P^40^. A High P-supply reduced root colonization, and the shift in the community structure of soil AMF was primarily associated with factors such as available P, N/P ratio, and soil pH, particularly in the topsoil layer^41^. The absence of any effect of compost on PSB also indicates that the chemical characteristics of the filter cake were not conducive to the flourishing of these bacteria.

Our study showed that the application of PSB, either without compost (T2) or with compost (T6), increased enzyme activity between 8 and 12 months after transplanting and increased the bacterial population (Fig. 1c). An increase in FDA in the bacterial treatment was consistent with the direct effect of PSB inoculation on the bacterial population and its activity. Previous studies have also reported a significant positive correlation between microbial enzyme activity and microbial biomass^42^. Furthermore, other reports have shown a positive effect of compost on microbial enzyme activity (FDA hydrolysis)^43^. Considering the concern of pathogenicity, inoculation with *P. dispersa* did not result in any visible disease symptoms; instead, it promoted sugarcane growth, indicating its beneficial role as a PGPB.

**Fig. 4:**
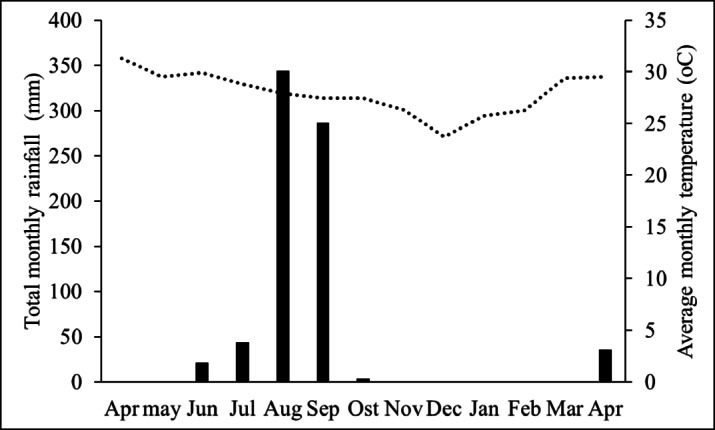
Average monthly temperature (line), and total monthly rainfall (bar) in Khon Kaen Province, Thailand from April 2019 to April 2020.

The physiological traits and growth (SPAD values, cane height, diameter, and tiller number) of sugarcane were improved by both fertilizer and biofertilizer applications. The combined treatment (T6) further enhanced growth and productivity compared to compost or microbial-only treatments, underscoring the synergistic effects of compost and microbial inoculation on plant performance. Moreover, T6 achieved growth and yield levels comparable to those of mineral fertilizer (T5), while also increasing soil microbial populations, activity, and soluble P in the rhizosphere, thus offering an added advantage over mineral fertilization. These results indicate that T6 is a viable alternative to conventional mineral fertilization, offering comparable yield performance while promoting a more environmentally friendly and sustainable sugarcane production system. Here, we noted a significant difference between the compost-only treatment (T4) and compost treatments with both guilds of soil microbes (T6). This difference indicated the additional contribution of these guilds to sugarcane performance. The important role of both guilds was also evident in comparisons between the control (T1) and the treatments with only PSB (T2) or AMF (T3), where significant yield increases were noted in both cases. Improvement of sugarcane plant performance due to AMF and PSB inoculation has been widely reported^32,44–46^, but the enhanced beneficial effect after the addition of filter cake compost has not been reported before. We interpret these effects as indicating that sugarcane performance is limited by P availability, because both the enhanced ability of the soil ecosystem to solubilize unavailable P and to take up available P enhanced growth (Table 1). However, alleviation of soluble P limitation^46,47^ could have resulted in larger plants that transpired more water and hence suffered more from drought. In this study, sugarcane plants frequently experienced water deficits, particularly during the grand growth stage, under natural field conditions (Fig. 4), which resulted in reduced plant performance as well as impaired growth and yield. The grand growth stage is most sensitive to water deficit, followed by the tillering stage^48^. Previous studies have reported yield reductions of 32–40% under rainfed conditions^49,50^. Inoculation and compost addition improved SPAD values, indicating enhanced photosynthetic efficiency, which was positively correlated with the photosynthesis rate and plant dry matter^51^. Although the beneficial effects of P on sugarcane are well established, K nutrition should also be considered. As shown in Table 1, available K was low relative to the beneficial levels of P, suggesting a potential K limitation. Low K availability can result from low cation exchange capacity, leaching (particularly in sandy soils), competition with other cations, and fixation within clay mineral layers. Both compost and mineral fertilizers substantially increased the total and available K in the rhizosphere. Maintaining a high K: P ratio with adequate P more than doubled yields compared with unfertilized controls while also improving juice quality. Fertilization with 180–240 kg K_2_O ha^−1^ increased soil K availability, improved plant uptake, and raised yields by up to 7.9%, while enhancing stalk size and weight^52,53^, as K activates enzymes involved in sucrose synthesis and translocation, promoting photosynthesis, sugar movement, and overall yield and quality^54^. These results suggest that the observed improvements in cane yield, °Brix, and CCS following bio-organic fertilizer application in both compost and mineral fertilizer treatments may reflect not only enhanced P availability but also improved K supply. PCA (Fig. 3) corroborates our univariate results by showing that T6 consistently groups with increased microbial activity, higher available P, and improved plant growth and productivity variables.

The application of PSB, AMF, or compost alone significantly improved sugarcane growth and productivity, although these effects were generally lower than those observed with mineral fertilizer (Table 2). The combined application of filter cake compost with PSB and AMF achieved growth and yield levels comparable to mineral fertilizer, while simultaneously enhancing soil microbial dynamics and increasing soluble rhizosphere P. Nevertheless, the high P content of this specific amendment warrants careful consideration, as it may influence interactions between organic amendments and beneficial microbial guilds. Further optimization of bio-organic amendment properties, particularly P levels, could help maximize the potential benefits for sugarcane growth.

## Conclusions

The addition of bio-organic fertilizer supplemented with AMF and PSB significantly enhanced sugarcane growth and productivity. The results demonstrated that this combined approach was more effective than mineral fertilizer or the individual application of organic amendments, AMF, or PSB. These findings suggest that integrating bio-organic amendments with microbial inoculants is a promising strategy to improve sugarcane cultivation in former paddy rice fields.

## Materials and methods

### (Bio-)fertilizer preparation

In a preliminary pot experiment under controlled greenhouse conditions with sterile soil, the PSB strain *P. dispersa* KKU-TP3 enhanced sugarcane growth, as shown by height, stalk diameter, leaf area, and chlorophyll content. Notably, it led to a significant 51% increase in plant P content, resulting in a 32% increase in plant dry weight compared with non-inoculated plants (unpublished observations). Consequently, this strain was selected for field trials. The PSB starter was cultured in nutrient broth at 150 rpm and 30 °C for 18 h. The inoculum was prepared in a 5 L Erlenmeyer flask using 2% (v/v) molasses in water as carbon source. The 3-liter culture medium was incubated with 2% (v/v) of PSB starter and then incubated at 150 rpm at 30 °C for 3 days. The culture was subsequently centrifuged at 8,000 rpm at 4 °C for 10 min to collect the bacterial cell pellets. The PSB inoculum was prepared by mixing cell pellets with twice sterilized rice husk charcoal to obtain a final bacterial concentration of approximately 10^8^ CFU g^−1^.

The AMF used was *Rhizophagus clarus* KKU-F3^55^. Mass production of AMF inoculum was conducted under greenhouse conditions by upscaling the pot culture technique at Khon Kaen University’s agronomic farm in Khon Kaen, Thailand. Maize seeds were surface sterilized with 10% sodium hypochlorite for 10 min and washed three times with sterile water. Seeds were transferred into sterile sand for germination in nursery pots for seven days. A sandy loam soil (pH 6.8; organic matter 2.7 g kg^−1^; total N 0.14 g kg^−1^; total P 0.05 g kg^−1^; total K 0.28 g kg^−1^) was sterilized twice by autoclaving at 121 °C, at a pressure of 15 psi (1.04 bar) for 15 min. 300 Kg twice-sterilized soil was placed into a planting bag box, 0.3 m height, 1.8 m long, and 0.6 m wide. Soil inoculum from 100 spores of *R. clarus* was inoculated into a planting hole (65 holes in total), and a healthy maize seedling was placed in the hole and filled with soil. Plants were watered with tap water once a day without additional mineral fertilizer for 90 days. Subsequently, the plants and soil were allowed to dry. Thereafter, the soil was crushed, and dry roots were separated, cut into segments, and mixed with the soil to be used as soil inoculum.

The filter cake was composted in an open space area for 3 months. The mature compost was then sieved and characterized for its properties. The seed germination index was used to assess compost phytotoxicity using the paper towel technique^56^. The properties of filter cake compost were: electrical conductivity 1.93 ds m^−1^, pH 8.28, organic matter 95.3 g kg^−1^, total N 6.0 g kg^−1^, total P 2.8 g kg^−1^, and total K 7.5 g kg^−1^. Chemical data and seed germination index (81%) indicated that the filter cake compost was mature with high amounts of plant nutrients and without phytotoxicity. This compost was used as the basal mixture, with or without PSB and AMF inocula. In the combined treatment of organic amendments, PSB, and AMF, a ratio of organic amendment: PSB: AMF of approximately 600: 335: 65 g kg^−1^ was used. The PSB inocula were premixed with the compost to achieve a final concentration of approximately 7.8 × 10^7^ CFU g^−1^ compost and allowed to air-dry to a moisture content of 15%. The AMF inoculum was then added to the premixed compost (with PSB suspension) to achieve a final spore concentration of approximately 5 spores per gram of compost.

A compound chemical fertilizer with a 15-15-15 NPK formulation was used for the mineral fertilizer treatment. The nutrients content of the fertilizer was approximately 115 g kg^−1^ of N from NH_4_NO_3_, 35 g kg^−1^ of N and 150 g kg⁻¹ of P (P_2_O_5_) from NH_4_H_2_PO_4_, and 150 g kg^−1^ of K (K_2_O) from KCl.

### Preparation of sugarcane seedlings and plantation

8-Month-old healthy sugarcane stalks of the KK3 cultivar were obtained from the Northeast Thailand Cane and Sugar Research Center, Khon Kaen University, Thailand. The stalks were cut into 5 cm long pieces, each containing one bud. Then, the sugarcane bud chips were rinsed with tap water and submerged in a 0.5% (v/v) of root supplement solution (T-REX B-1 start, Thailand) in tap water for 1 h. Sugarcane bud chips were planted in 0.6 × 10 cm plastic bags containing rice husk charcoal and filter cake compost at a ratio of 607:393 g kg^−1^. Sugarcane seedlings were nursed in a shade net house for 45 d. Leaf clipping was performed, involving the cutting of seedling leaves to leave approximately 7–8 cm to reduce transpiration before transplanting them to the field.

### Experimental design and crop management

The experimental field was located in the Ban Fang District, Khon Kaen Province, Thailand (Latitude: 16.456857 N; Longitude: 102.617447 E). The site had a long history of continuous rice cultivation for several decades; however, frequent droughts often caused crop failure or very low yields. The soil was classified as sandy loam with a moderate depth and was prone to erosion and gully formation. Soil fertility was low, with an organic matter content < 15 g kg^−1^, available phosphorus < 10 mg kg^−1^, and available K < 60 mg kg^−1^. The cation exchange capacity was < 10 cmol kg^−1^, with base saturation < 30% [^57^ (in Thai)]. The field has long been managed predominantly with chemical fertilizers, along with routine herbicide and insecticide applications, although inputs vary across cropping seasons under farmer management.

The field experiment was conducted from April 2019 to April 2020. The average monthly temperature and total monthly rainfall during the study period are shown in Fig. 4. The experiment was carried out using a randomized complete block design (RCBD) with 6 treatments and 4 replications. The treatments were T1 (control): plants received neither fertilizer nor inoculum; T2: plants were inoculated with PSB at a rate of 202 kg ha^−1^, each containing over 15.2 × 10^8^ CFU plant^−1^ and nutrient contribution from PSB carrier ≈ 0.03 kg N, 0.01 kg P, 0.06 kg K ha^−1^; T3: plants were inoculated with AMF at a rate of 1048 kg ha^−1^, each containing over 1400 spores plant^−1^ and nutrient contribution from AMF carrier ≈ 0.15 kg N, 0.05 kg P, 0.29 kg K ha^−1^; T4: plants received filter cake compost at a rate of 1875 kg ha^−1^, supplying ≈ 11.25 kg N, 5.25 kg P, and 14.06 kg K ha^−1^; T5: plants received mineral fertilizer at a rate of 312.5 kg ha^−1^, supplying ≈ 46.88 kg of each N, P, and K ha^−1^; T6: plants received filter cake compost supplemented with AMF and PSB at a rate of in total 3125 kg ha^−1^, supplying in total ≈ 11.43 kg N, 5.31 kg P, and 14.41 kg K ha^−1^.

Land preparation was performed according to the standard procedure for sugarcane cultivation. Primary plowing was performed at a depth of 25–30 cm. After 10–15 d, secondary plowing or harrowing was conducted to break up the soil clods and create a finer seedbed. Planting furrows or ridges were then formed. Each plot, with a size of 6 × 7.5 m (45 m^2^), consisted of 5 rows with a spacing of 1.5 m between rows and 0.5 m between plants. Each plot had a co-buffer area consisting of 1 row and 2 m at the ends of each row. Sugarcane seedlings were transplanted to the field with a plot set at approximately 20 cm deep. Before transplantation, water was added to the field soil to provide sufficient moisture.

PSB, AMF, filter cake compost, mineral fertilizer, and filter cake compost mixed with PSB and AMF were applied in two equal splits: the first half was placed beneath the root ball at transplanting, and the second half was applied beside the clumps four months later, and then incorporated into the soil by plowing. To ensure that the observed effects could be attributed primarily to the applied amendments and inocula, weeds were removed manually, and no insecticides were applied throughout the cropping period.

### Determination of microbial dynamics in soils

Microbial dynamics were assessed at 4, 8, and 12 months after transplantation, using samples collected from the three middle rows of each plot. Soil samples, including roots, were randomly taken from the rhizosphere of 10 plants per plot using a 15 cm open-face auger at a depth of 0–20 cm away from the soil surface. Fresh samples were thoroughly mixed before sub-sampling to determine the bacterial populations and microbial activity. Subsequently, sub-samples were air-dried and passed through a 2 mm sieve for mycorrhizal assessment.

To determine AMF root colonization and the number of AMF spores, sugarcane rhizosphere soils were air-dried before separating the roots and soil. The roots were stained according to a previously described method^58^. Root samples were washed using tap water, cleared in a 2.5% KOH solution for 1 h at 90 °C, subsequently acidified with 1% HCl solution before staining for 8 h with 0.05% trypan blue in acetic glycerin solution. The AMF colonization levels were observed under a stereomicroscope (Nikon Eclipse 50i, Japan) and scored based on the method described by Trouvelot et al.^59^. The quantity of AMF spores was determined from dried rhizosphere soils according to Daniels and Skipper^60^. Briefly, soil was suspended in tap water and centrifuged at 4,000 rpm for 1 min to discard the supernatant. AMF spores were isolated from the soil by thorough mixing with 50% sucrose solution, then centrifuged at 4,000 rpm for 5 min. The supernatant was filtered through a sieve (45 μm pore diameter). AMF spores left on the sieve were washed with distilled water before being transferred onto filter paper in a Petri dish for counting under a stereomicroscope (Nikon Eclipse 50i, Japan).

Viable bacteria were enumerated using the standard plate count method. Fresh soil samples were resuspended in sterile distilled water after 10-fold serial dilution before plating onto plate count agar. Plates were incubated at room temperature for 48 h. Bacterial colonies were counted, and the number of CFUs per gram of soil was calculated.

The microbial enzyme activity was determined using the FDA analysis method described by Green et al.^61^. Fresh soil samples (1 g) were mixed with 7.5 ml of 60 mM sodium phosphate buffer and 0.1 ml of FDA (1 mg ml^−1^ stock solution dissolved in acetone). The reaction mixture was incubated at room temperature with shaking at 150 rpm for 30 min. Further extraction was performed by vigorously mixing a chloroform and methanol (2:1) solution (7.5 ml). The solution was then centrifuged at 10,000 rpm for 10 min to collect the top layer solution. An extract of the autoclaved soil reaction mixture was used as a blank control. Samples were assayed for FDA hydrolysis at a wavelength of 490 nm. Microbial activity was calculated by comparison with the fluorescein standard curve.

### Determination of plant growth parameters

Plant growth parameters, including height, stem diameter, tillering, and SCMR, were measured 4, 8, and 12 months after transplantation, with samples collected from the three middle rows of each plot. Ten plants were located at the same points as the soil samples in each plot. Plant height was measured from the ground to the insertion of the top visible dewlap leaf (TVD), using a meter ruler. The stalk diameter was determined by averaging the measurements taken at the bottom, middle, and top of the stalks using a Vernier caliper. Tillering was assessed by counting the number of stalks per plant. SCMR was examined in leaves below the insertion of the TVD. SCMR, an indirect indicator of chlorophyll content, was measured at 9–10 a.m. on the sampling day using a SPAD meter (SPAD-502, Konica Minolta, Japan).

### Determination of nutrients in the rhizosphere

At the end of the experiment (12 months after transplanting), soil samples were randomly collected in the vicinity of the root zone from 10 plants of individual plots, mixed, air-dried, and passed through a 2 mm sieve for determination of nutrients. Soil chemical properties, including total organic matter, total N, P, and K, and available P and K contents, were investigated by the research unit service at the Department of Plant Science and Agricultural Resource, Faculty of Agriculture, Khon Kaen University. The method used was carried out following the method of Flow Injection Analysis (FIA) after Kjeldahl digestion for N^62^. Organic matter was assessed using the wet oxidation method described by Walkley and Black^63^. Total P and K were extracted using a wet oxidation method with nitric acid and perchloric acid (2:1, v/v). Total P was followed by colorimetric determination using the molybdenum blue method, measuring absorbance with a spectrophotometer at 820 nm. Total K was measured using the flame photometer method at 768 nm^64^. Available P in the soil was determined using Bray–II according to Bray and Kurtz^65^. Exchangeable K was determined using exchangeable cations in the soil. Briefly, the soil was extracted using 1 N NH_4_OAc, then measured using the flame photometer method at 589 nm^66^.

### Determination of sugarcane productivity

Sugarcane plants were randomly collected within a 2 m^2^ area after 12 months. Cane fresh yield was measured when plants were manually harvested. All stalks from each plot were cut from the ground to TVD using hand knives, and leaves were subsequently stripped off from the stalk. Cane stalks were immediately weighed with 10 stalks at a time using a 60 kg dial spring balance (LEO, Thailand) to record the total weight of the cane stalks in the plot area. Cane yield was calculated as t ha^−1^ using Eq. (1):

1 $$\:{\text{Cane}}\:{\text{yield}}\:({\text{t}}\:{\text{ha}}^{{ - 1}} {\text{) = }}\frac{{{\text{10}},{\text{000}}\: \times \:{\text{total}}\:{\text{weight}}\:{\text{of}}\:{\text{cane}}\:{\text{stalks}}\:\left( {\text{t}} \right)}}{{{\text{harvest}}\:{\text{area}}\:\left( {{\text{m}}^{{\text{2}}} } \right)}}$$

Subsequently, cane stalks were randomly collected with 10 stalks at a time to measure the amount of dissolved solids in a liquid solution (°Brix or %Brix) and CCS percentages. On harvest day, the Brix value was immediately estimated by refractometry using a digital Brix meter (pocket refractometer PAL-1, Japan). The CCS percentage was measured using traditional methods conducted by the research unit at the Khon Kaen Field Crop Research Center, Khon Kaen, Thailand. The CCS value was then calculated from the percentage by weight of sucrose dissolved in sugarcane juice (%Pol), percentage by weight of total dissolved solids in sugarcane juice (%Brix), and percentage of sugarcane fiber (%Fiber) according to Eq. (2)^67^:

2 $$\:{\text{CCS = }}\frac{{{\text{0}}{\text{.9433}}\left( {\% {\text{Pol}}} \right)\left( {{\text{100}} - \% {\text{Fiber}}} \right)}}{{{\text{100}}}} - \frac{{\text{1}}}{{\text{2}}}\left( {\frac{{{\text{(0}}{\text{.9660}}\left( {\% {\text{Brix}}} \right))({\text{100}} - \% {\text{Fiber}})}}{{{\text{100}} - {\text{0}}.{\text{9433}}(\% {\text{Pol}})\frac{{({\text{100}} - \% {\text{Fiber}})}}{{{\text{100}}}}}}} \right)$$

### Statistical analysis

Data on the growth and physiology of sugarcane, microbiological soil attributes, yields of sugarcane, and nutrient content in soil and plants were subjected to one-way analysis of variance (ANOVA) with experimental RCBD design, followed by Tukey’s Honestly Significant Difference (HSD) test at *P* ≤ 0.05, using Statistix 8.0 program. The mean values were reported based on four replicates, along with standard deviations. PCA was performed using the OriginPro 2025b program to evaluate the relationships between treatments on measured parameters. Prior to PCA, data suitability was confirmed through the Kaiser–Meyer–Olkin (KMO) measure of sampling adequacy and Bartlett’s test of sphericity. Variables with communality values < 0.5 were excluded, and principal components were retained based on the eigenvalues > 1.0.

## Data Availability

Data will be made available upon reasonable request by contacting Suchat Juntahum.
